# Supplementary material for: Multi‐dataset identification of innovative feature genes and molecular mechanisms in keratoconus
Source: J Cell Mol Med. 2024 Sep 19;28(18):e70079. doi: 10.1111/jcmm.70079 (PMC11412914; doi:10.1111/jcmm.70079)
Supplement: Supplementary file 2 — Material S2. [file JCMM-28-e70079-s002.zip › 7.GSEA analysis/2.ATOH7/KEGG_PENTOSE_AND_GLUCURONATE_INTERCONVERSIONS.html]

Details for gene set KEGG\_PENTOSE\_AND\_GLUCURONATE\_INTERCONVERSIONS[GSEA]

|  || Dataset | ATOH7.group.cls#h\_versus\_l |
| Phenotype | group.cls#h\_versus\_l |
| Upregulated in class | l |
| GeneSet | KEGG\_PENTOSE\_AND\_GLUCURONATE\_INTERCONVERSIONS |
| Enrichment Score (ES) | -0.4855886 |
| Normalized Enrichment Score (NES) | -1.2824578 |
| Nominal p-value | 0.14439656 |
| FDR q-value | 1.0 |
| FWER p-Value | 0.953 |
Table: GSEA Results Summary

  

Fig 1: Enrichment plot: KEGG\_PENTOSE\_AND\_GLUCURONATE\_INTERCONVERSIONS      
 Profile of the Running ES Score & Positions of GeneSet Members on the Rank Ordered List

  

| SYMBOL | TITLE | RANK IN GENE LIST | RANK METRIC SCORE | RUNNING ES | CORE ENRICHMENT || 1 | UGT2B7 | na | 1726 | 0.367 | 0.0587 | No |
| 2 | AKR1B1 | na | 3374 | 0.261 | 0.0884 | No |
| 3 | XYLB | na | 5674 | 0.180 | 0.0754 | No |
| 4 | UGT2B15 | na | 6980 | 0.144 | 0.0803 | No |
| 5 | UGT2A1 | na | 8781 | 0.103 | 0.0588 | No |
| 6 | DHDH | na | 9681 | 0.086 | 0.0584 | No |
| 7 | UGDH | na | 12373 | 0.048 | -0.0050 | No |
| 8 | UGT2B11 | na | 13663 | 0.034 | -0.0321 | No |
| 9 | RPE | na | 14817 | 0.023 | -0.0586 | No |
| 10 | UGP2 | na | 14844 | 0.023 | -0.0527 | No |
| 11 | CRYL1 | na | 15069 | 0.021 | -0.0531 | No |
| 12 | GUSB | na | 16038 | 0.013 | -0.0773 | No |
| 13 | UGT2A3 | na | 16675 | 0.007 | -0.0934 | No |
| 14 | UGT2B10 | na | 19029 | -0.013 | -0.1573 | No |
| 15 | DCXR | na | 22739 | -0.051 | -0.2490 | No |
| 16 | UGT2B4 | na | 30951 | -0.245 | -0.4131 | Yes |
| 17 | UGT1A6 | na | 33234 | -0.388 | -0.3643 | Yes |
| 18 | UGT2B28 | na | 33254 | -0.390 | -0.2495 | Yes |
| 19 | RPEL1 | na | 33491 | -0.415 | -0.1337 | Yes |
| 20 | UGT1A8 | na | 34302 | -0.573 | 0.0123 | Yes |
Table: GSEA details [plain text format]

  

Fig 2: KEGG\_PENTOSE\_AND\_GLUCURONATE\_INTERCONVERSIONS      
 Blue-Pink O' Gram in the Space of the Analyzed GeneSet

  

Fig 3: KEGG\_PENTOSE\_AND\_GLUCURONATE\_INTERCONVERSIONS: Random ES distribution      
 Gene set null distribution of ES for **KEGG\_PENTOSE\_AND\_GLUCURONATE\_INTERCONVERSIONS**
